# Familial homozygous hypercholesterolemia with arcus cornea and xanthomas: A rare but serious entity

**DOI:** 10.1002/ccr3.7024

**Published:** 2023-03-04

**Authors:** Amal Chamli, Anissa Zaouak, Refka Frioui, Samy Fenniche, Houda Hammami

**Affiliations:** ^1^ Dermatology Department Habib Thameur Hospital Tunis Tunisia; ^2^ Faculty of medicine of Tunis, University of Tunis El Manar Tunis Tunisia

**Keywords:** cornea, familial hypercholesterolemia, homozygous, hypercholesterolemia, xanthomatosis

## Abstract

Familial hypercholesterolemia (FH) is a rare but life‐threatening disorder. Skin manifestations can be its only manifestation. We present a case of a fifteen‐year‐old female child, with multiple eruptive xanthomas, xanthomas anarcus, and a deranged lipid profile consistent with FH. The presence of this manifestation especially in the younger age group should draw attention to hypercholesterolemia. A timely diagnosis is fundamental to prevent serious complications and for early treatment.

## Abstract 2

We present a case of fifteen‐year‐old female child, with eruptive xanthomas, xanthomas anarcus, and deranged lipid profile consistent with FH. The presence of this manifestation especially in the younger age group should draw attention to familial hypercholesterolemia. A timely diagnosis is fundamental to prevent serious complications and for early treatment.

## 1 CASE PRESENTATION

Familial hypercholesterolemia (FH) is a rare but life‐threatening disorder. Skin manifestations can be its only manifestation. We present a case of a fifteen‐year‐old female child, with multiple eruptive xanthomas, xanthomas anarcus, and a deranged lipid profile consistent with FH.

**FIGURE 1:**
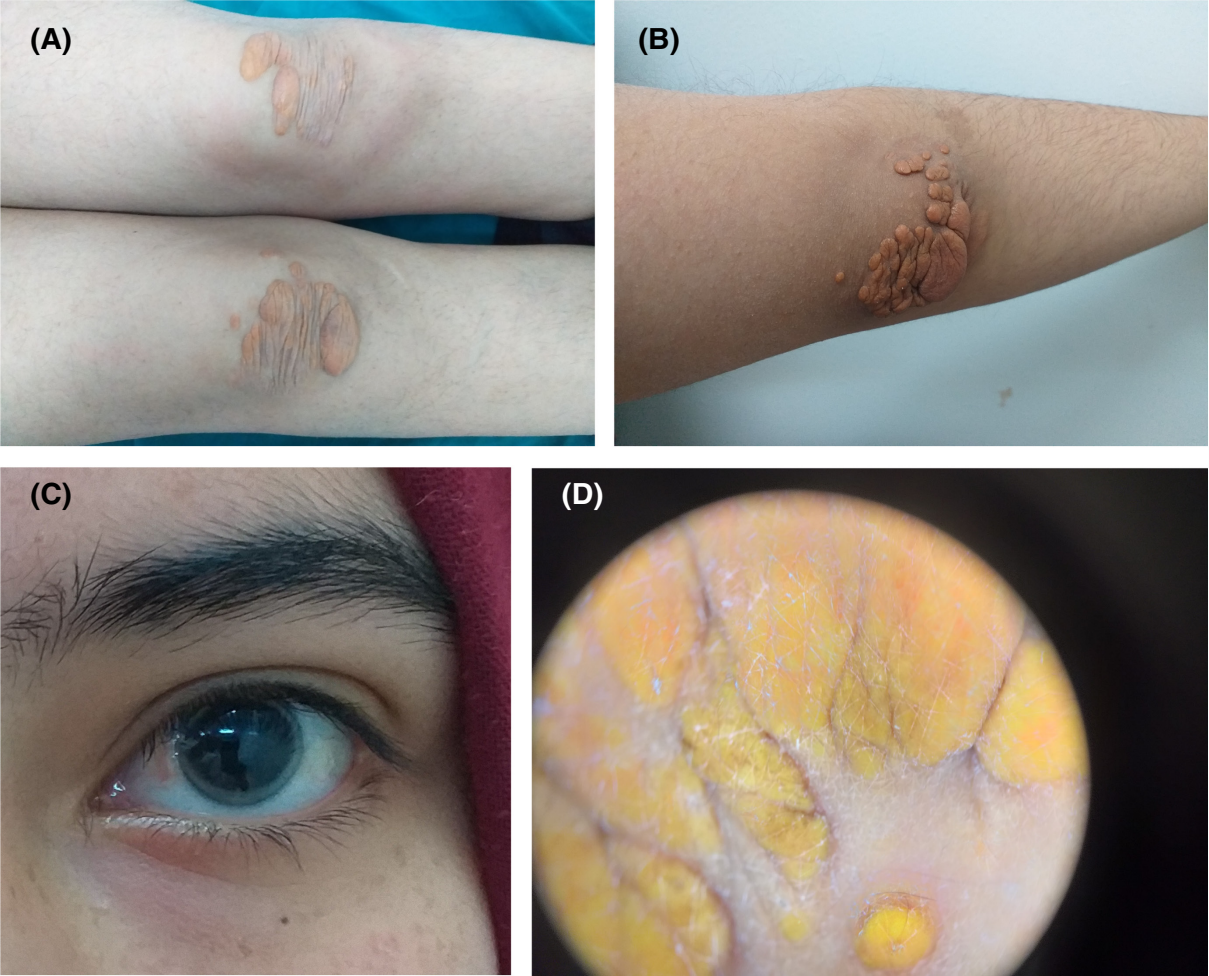
(A) Multiple yellowish plaques over the knees, (B) Multiple yellowish papulo‐nodules on the right elbow, (C) Corneal arcus, (D) Dermoscopy of the right elbow showing multiple regularly shaped yellowish areas (x20).

**FIGURE 2:**
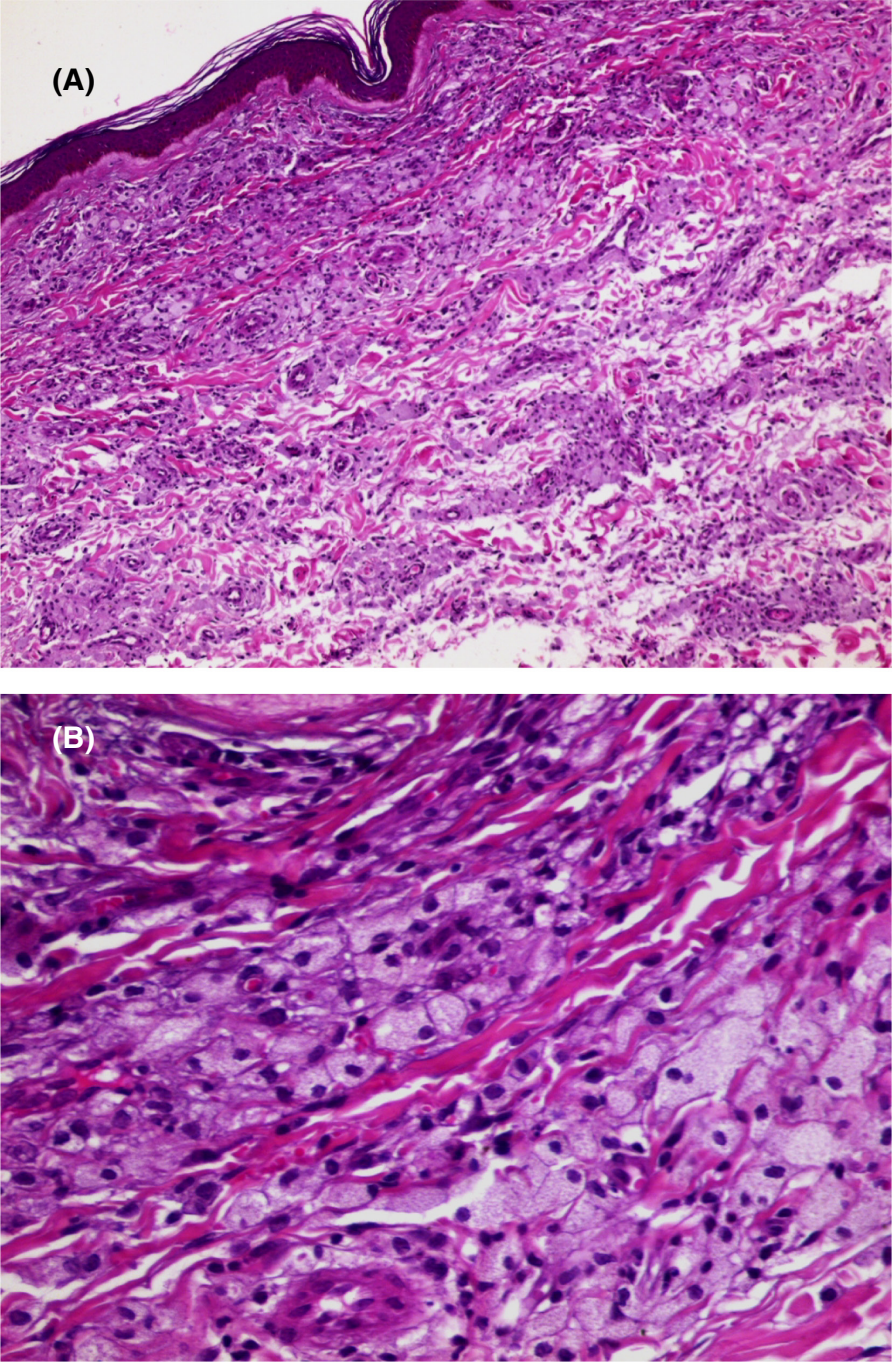
(A) Under a normal epidermis, presence of a proliferation of foam cells in the dermis within collagen bundles (HEX100), (B) Proliferation of foam cells in the dermis (HEX400).

A 12‐year‐old girl born to non‐consanguineous marriage presented to our dermatology department with generalized asymptomatic skin lesions evolving for 3 years. On examination, there were yellowish, non‐tender, mobile, papules on an erythematous base over the arms, thighs, buttocks, soles. They were up to 6 cm in size. The lesions involved the bilateral elbows, knees, in addition to firm subcutaneous nodules at Achilles’ tendon (Figure 1A,B). Ophthalmic examination showed corneal arcus (Figure 1C). Dermoscopy revealed a homogeneous yellow color, corresponding to dermal xanthomatous deposits, with interconnected fine and dotted vessels (Figure 1D). Complete blood count, liver, renal, and thyroid function tests were within normal limits. The laboratory findings showed normal levels of triglycerides (84.42 mg/dL), an elevated levels of total cholesterol (576 mg/dL) and low‐density lipoprotein cholesterol (LDL‐c) (432 mg/dL), and low high‐density lipoprotein (HDL) (30 mg/dL). A skin biopsy demonstrated foamy cells, with extracellular lipid between collagen bundles in the dermis (Figure 2A,B). Based on this finding, a diagnosis of homozygous familial hypercholesterolemia was made. Treatment was started with statins and resins. The patient was further referred for cardiologic and angiological examinations. Lipid profile screening was advised for all the family. Family history of similar condition was positive in elder brother (16 years of age). There was no history of myocardial infraction in the the family members.

## 2 DISCUSSION

Familial hypercholesterolemia (FH) is an autosomal dominant inherited disorder of lipid metabolism. Untreated FH may lead to persistent hypercholesterolemia and therefore life‐threatening conditions such as cardiovascular disorders. Heterozygote and homozygote types have been described.^1^ In our patient, the lipid profile was suggestive of homozygous type. Homozygous type is rare and presents in early childhood with a high risk of early onset of atherosclerosis and premature coronary death, whereas heterozygous type is the most common and becomes symptomatic in the third or sixth decade.^1^ Clinical diagnosis is often straightforward since in persistent hypercholesterolemia, skin manifestations appear as xanthomas or arcus cornea. Xanthomas are considered a classical sign of FH. Their appearance in children and adolescents is more suspicious of a severe form of hypercholesterolemia.^2^ Clinically, xanthomas appear as yellow papules, plaques, or nodules due to abnormal deposition of lipid in the foam cell and collagen. There are different aspects of xanthomas depending on their clinical manifestations. Tendon xanthomas are firm subcutaneous nodules and are localized over the Achilles, patellar tendons, and extensor tendons of the hands. Other types are eruptive xanthomas involving extensor surfaces, trunk, as well as gluteal region, as in our patient.^2^, ^3^ Furthermore, arcus cornea is an important sign of FH and clinically appears as a single grayish ring parallel to the limbus. Its prevalence increases with age, and it is caused by lipid deposition in the deep corneal stroma and the limbal sclera.^4^

Sometimes, skin manifestations can be the only manifestation of an underlying lipid abnormality, as in our patient. Interestingly, xanthomas lead patients with homozygous FH to consult dermatologists or plastic surgeons first because of its aesthetic aspect. Thus, the presence of xanthomas or arcus cornea, especially in the younger age group, should draw attention to hypercholesterolemia. A timely diagnosis is fundamental to prevent serious complications and for early treatment.

## AUTHOR CONTRIBUTIONS

Dr. Amal Chamli is the guarantor of the content of the manuscript, included the data and analysis. Dr. Refka Frioui contributed to interpretation of data and revision of the manuscript. Dr. Anissa Zaouak, Dr Houda Hammami, and Dr Samy fenniche contributed to analysis and interpretation of data, revised it critically for important intellectual content, and final approval of the version to be submitted.

## FUNDING INFORMATION

None.

## CONFLICT OF INTEREST STATEMENT

The authors declare that there are no conflicts of interest in this work.

## ETHICAL APPROVAL

Informed consent was obtained from the patient.

## CONSENT

The examination of the patient was conducted according to the principles of the Declaration of Helsinki. The authors certify that they have obtained all appropriate patient consent forms, in which the patient gave his consent for images and other clinical information to be included in the journal. The patient understood that his name and initial will not be published and due effort will be made to conceal his identity, but that anonymity cannot be guaranteed.

## Data Availability

Not applicable.
